# Fundal Height Growth Curve for Thai Women

**DOI:** 10.1155/2013/463598

**Published:** 2013-04-15

**Authors:** Jirawan Deeluea, Supatra Sirichotiyakul, Sawaek Weerakiet, Renu Buntha, Chamaiporn Tawichasri, Jayanton Patumanond

**Affiliations:** ^1^Clinical Epidemiology Program, Faculty of Medicine, Chiang Mai University, Chiang Mai 50200, Thailand; ^2^Department of Obstetrics and Gynecology, Faculty of Nursing, Chiang Mai University, Chiang Mai 50200, Thailand; ^3^Department of Obstetrics and Gynecology, Faculty of Medicine, Chiang Mai University, Chiang Mai 50200, Thailand; ^4^Department of Obstetrics and Gynecology, Faculty of Medicine, Ramathibodi Hospital, Mahidol University, Bangkok 10400, Thailand; ^5^Antenatal Care & Family Planning Clinic, Phayao Hospital, Phayao 56000, Thailand; ^6^Clinical Epidemiology Unit, Faculty of Medicine, Chiang Mai University, Chiang Mai 50200, Thailand

## Abstract

*Objectives*. To develop fundal height (FH) growth curve from normal singleton pregnancy based on last menstrual period (LMP) and/or ultrasound dating for women in the northern part of Thailand. *Methods*. A retrospective time-series study was conducted at four hospitals in the upper northern part of Thailand between January 2009 and March 2011. FH from 20 to 40 weeks was measured in centimeters. The FH growth curve was presented as smoothed function of the 10th, 50th, and 90th percentiles, which were derived from a regression model fitted by a multilevel model for continuous data. *Results*. FH growth curve was derived from 7,523 measurements of 1,038 women. Gestational age was calculated from LMP in 648 women and ultrasound in 390 women. The FH increased from 19.1 cm at 20 weeks to 35.4 cm at 40 weeks. The maximum increase of 1.0 cm/wk was observed between 20 and 32 weeks, declining to 0.7 cm/wk between 33 and 36 weeks and 0.3 cm/wk between 37 and 40 weeks. A quadratic regression equation was FH (cm) = −19.7882 + 2.438157 GA (wk) − 0.0262178 GA^2^ (wk) (*R*-squared = 0.85). *Conclusions*. A demographically specific FH growth curve may be an appropriate tool for monitoring and screening abnormal intrauterine growth.

## 1. Introduction

Routine symphysis-fundal height (or “fundal height” in short) measurement during pregnancy has been used in antenatal care with a long history, to estimate size of uterus and gestational age. It is simple, convenient, safe, and cheap [1–4]. Abnormal fundal height (smaller or larger than gestational age) may indicate abnormal uterus, fetal growth, and amniotic fluid development. Fundus smaller than gestational age may indicate intrauterine growth restriction (IUGR), small for gestational age (SGA), or oligohydramnios, while fundus larger than gestational age may reflect large fetus for gestational age (LGA), polyhydramnios, twins, or uterine tumor [5].

Although ultrasound is replacing fundal height measurement in detecting the above conditions [6, 7], in developing countries, it is not fully available in all antenatal care levels, due to high cost and lack of experienced personnel [1, 3]. Therefore WHO Reproductive Health Library still recommends using fundal height measurement as a tool to estimate gestational age and detect SGA and multiple pregnancies [1]. National Institute for Health and Clinical Excellence Guideline for Antenatal Care (clinical guideline 62) also recommends routine measurement and monitoring fundal height for every antenatal visit [8].

According to Cochrane review, there is not enough evidence to evaluate the use of fundal height measurement during antenatal care [2]; on the other hand, there is also insufficient evidence to determine whether fundal height measurement is not effective [9]. The sensitivity of fundal height measurement in detecting IUGR, SGA, and LGA varies from 17% to 86% [10, 11], due to differences in gestational age assessment and fundal height measurement techniques [3]. Many studies therefore recommend demographically specific fundal height growth curve rather than universally derived curve, as fundal height is influenced by ethnicity, socioeconomics, and nutritional status [12–15].

Fundal height growth curves in Thai women were derived from specific setting with different subject selection and gestational age assessment [16, 17]. Most settings were university hospitals located in Bangkok, while the majority of pregnant women attended the general hospitals or primary care settings and most of them were from middle to low economic status. Gestational age assessment by last menstrual period (LMP) in the past [16] was replaced by ultrasound in more recent studies [17]. Although ultrasound is more accurate during the first half of pregnancy [7], both methods are still applied in most antenatal care services.

We developed fundal height growth curve from normal singleton pregnancy in general hospitals, using gestational age assessment both from LMP and/or ultrasound to reflect routine antenatal care practice. The derived fundal height growth curve is expected to be used in the northern part of Thailand.

## 2. Subjects and Methods

### 2.1. Subjects

This was the retrospective time-series study. All data were obtained from Thai women who attended antenatal care and delivered at four university affiliated hospitals in the upper northern part of Thailand, two provincial hospitals and two regional hospitals, between January 2009 and March 2011. Being administered under the Ministry of Public Health of Thailand, the four hospitals have a similar guideline for antenatal care practice. Subject eligible criteria were normal singleton pregnant women who started antenatal care before 20 weeks of gestation. Pregnant women with uncertain gestational age and medical or obstetrical complications affecting fetal growth and those with habitual smoking, alcohol drinking, and drug abuse during pregnancy were excluded from the study.

### 2.2. Ascertainment of Gestational Age

Gestational age was calculated from (1) first day of LMP if regular menstruation and correlated with size of uterus by palpation or correlated with gestational age by ultrasound (not more than 1 week difference) or (2) ultrasound in the first half of pregnancy if LMP uncontained or size of uterus not correlated with LMP or gestational age by LMP not correlated with ultrasound (more than 1 week difference).

### 2.3. Fundal Height Measurement

Fundal height was measured in centimeters with nonelastic measurement tape from the upper border of the symphysis pubis to the top of the uterine fundus, or reversed direction. All measurements were performed by or under supervision of registered nurses or obstetricians who had at least 2 years of experience with obstetric prenatal care, in order to minimize measurement error and bias [21].

### 2.4. Data Collection

Fundal height and gestational age were recorded from the beginning to the end of antenatal care. Labor notes and medical records were reviewed for relevant information.

### 2.5. Statistical Analysis

Assessment of gestational age and measurement of fundal height were standardized by a correction factor calculated from systematic error by a regression technique. A second-degree polynomial equation was fitted using a multilevel model for continuous data. A quadratic regression model was used to predict the 10th, 50th, and 90th percentiles of fundal height. The final quadratic regression model was applied to smooth each percentile line. The fundal height growth curve was presented as smoothed function of the 10th, 50th, and 90th percentiles between 20 and 40 weeks of gestation. All analysis was done using a standard statistical software package. 

### 2.6. Ethical Approval

The study protocol was approved by the Research Ethics Committee, Faculty of Medicine, Chiang Mai University, and the research ethics committee of the four hospitals.

## 3. Results

During the study period, there were 2,351 pregnant women who attended antenatal care and delivered at the four hospitals. Normal singleton pregnancies met with eligible criteria in 1,038 subjects. Among these, 696 (67.0%) were from provincial hospitals and 342 (33.0%) from regional hospitals. The proportion of eligible subjects ranged from 20.3% to 60.7%. Characteristics of the study subjects in the provincial and regional hospitals were similar. The average age was 25.6 years (SD = 6.2), body mass index (BMI) was 21.6 kg/m^2^ (SD = 3.8), and gestational age at first antenatal visit was 13 weeks (SD = 5.0). The proportion of nulliparity and multiparity were similar. The average of total pregnancy weight gain was 13.5 kg (SD = 4.7), gestational age at delivery was 39.2 weeks (SD = 1.1), and birth weight was 3,120.3 g (SD = 325.0) (Table 1).

Gestational age was calculated from LMP in 648 women (62.4%) and from ultrasound in 390 women (37.6%). Ultrasound was done at the average gestational age of 16 weeks (SD = 5.2). A total of 7,634 fundal height (FH) measurements were used with 111 missing data (1.4%). The remaining 7,523 measurements (98.6%) were analyzed, averaged 7.3 measurements per woman (SD = 2.1) and 358.2 measurements per week (range: 119–840).

**Figure 1:**
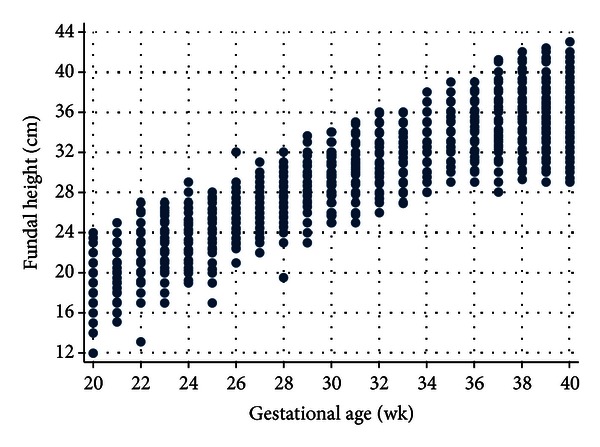
The scatter plot of fundal height (cm) for each gestational age (wk) based on 1,038 normal singleton pregnancies (7,523 visits).

The fundal height was likely to be equally distributed across each gestational age (GA) with an obvious monotonous increment from 19.1 cm (SD = 1.9) at 20 weeks to 35.4 cm (SD = 2.4) at 40 weeks (Table 2 and Figure 1). The average increase per week was 0.8 cm. The maximum increase of 1.0 cm/wk was observed between 20 and 32 weeks, declining to 0.7 cm/wk between 33 and 36 weeks and 0.3 cm/wk between 37 and 40 weeks (Table 2).

The fundal height obtained from quadratic regression equation allowing for random (individual) effect;

(1) FH (cm)=−19.7882+2.438157 GA (wk)−0.0262178 GA2 (wk).

The above equation explained 85% of the variation (*R*-squared = 0.85).

**Figure 2:**
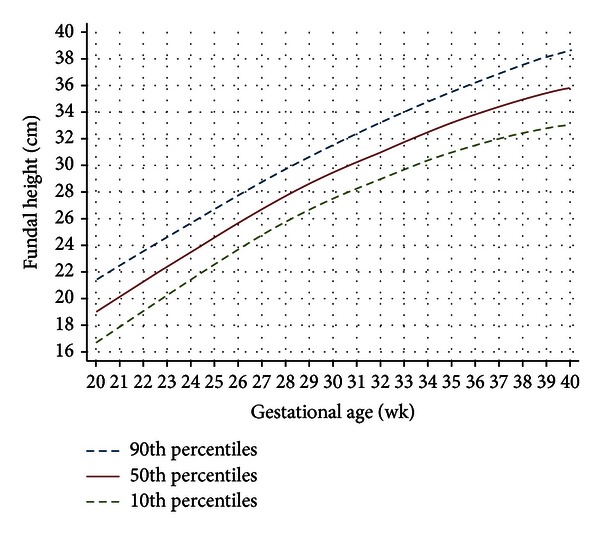
Fundal height growth curve at the 90th, 50th, and 10th percentiles based on 1,038 normal singleton pregnancies (7,523 visits).

The final fundal height growth curve (Figure 2) was presented as smoothed function of the 10th, 50th, and 90th percentiles derived from Table 3.

## 4. Discussion

Our fundal height growth curve was different from previous studies (Table 4) in the aspect of week-specific value, slope, and curve pattern [12–20]. These could be due to differences in ethnicity, socioeconomic, life style, nutritional status, study methodology, eligible criteria, gestational age definition, fundal height measurement, and statistical analysis. Our growth curve has a quadratic pattern similar to studies in Thailand [16, 17], Tanzania [13], and Nigeria [15] but was different from studies in Sweden [12] and Mozambique [14] which had cubic pattern curves. Thai women are relatively smaller and have relatively smaller pelvis compared to Caucasian women [22], causing an increase in a fundal height early in pregnancy (Figure 2). Enlargement of fundal height in Caucasian women with relatively larger and broader pelvis [23] was noticeable later in pregnancy, causing an S-shaped (cubic curve). A decline in fundal height around term was caused mainly by fetal engagement. Black women with African ethnicity with anthropoid typed pelvis (long anteroposterior diameter, short transverse diameter) [24] had similar fundal height pattern of growth curve to Thai women but did not decline around term due to unengagement [15]. An exception was observed in some African ethnic with gynecoid pelvis [25].

**Figure 3:**
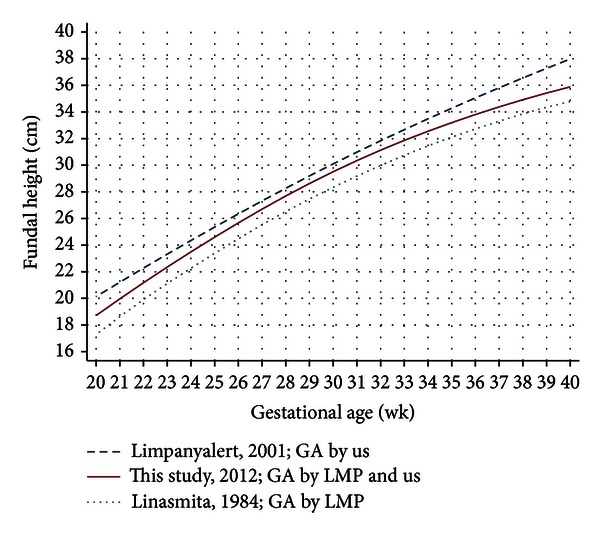
Fundal height growth curves at the 50th percentile derived from Thai women.

Confined to fundal height growth curve in Thai women, our curve is 1.0 cm above the study in 1984 [16], but 0.5 to 1.0 cm below the study in 2001 [17] (Figure 3). The discrepancies may reflect difference population and/or study methodology, including the cohort effect. The fact that mean birth weight in the northern part of the country increased from 2,933 g in 1982 [26] to 3,117 g in 2011 [27] may explain the latter hypothesis.

It is worth noticing that gestational age in three studies used different criteria (Figure 3). Although gestational age by LMP tends to overestimate ultrasound [28, 29], our study had explored and confirmed that gestational age by LMP and by ultrasound was very close. Errors in ultrasound are less in women with low BMI [30]. The fact that our subjects had an average BMI 21.6 kg/m^2^ (SD = 3.8) may explain the above statement.

As mentioned above, it is therefore essential that each population should have its own fundal height growth curve to use in screening for abnormal intrauterine growth. Event in the same country, different context of ethnicity, and socioeconomic, measurement method also lead to difference in fundal height growth curve. Our study focuses on development of fundal height growth curve based on routine antenatal care practice in the northern part of Thailand, which may be different from the rest of the country.

The regression technique in our study considered correlation of fundal height and gestational age within the same subject. A multilevel model for continuous data using longitudinally collected data is more appropriate than a cross-sectionally collected data in some studies. Observable larger variation of fundal height early and late pregnancy (Figure 1) may be obscured by thick abdominal wall especially in early pregnancy and fetal engagement beyond 37 weeks.

Existing fundal height growth curve used difference criteria: lower-upper limit, ±1 to ±2 standard deviation, and the 10th to 90th percentile or 5th to 95th percentile. We chose the 10th to 90th percentile to focus on screening rather than diagnosis. We also recommend monitoring fundal height at every antenatal visit. Medical consultation or further investigation is recommended when fundal height is below the 10th, or above 90th percentile; fundal height growth rate decelerates, stabilizes or declines, or increases rapidly. 

Screening for abnormal uterine growth and gestational age in the past assumed a constant linear equation; FH (cm) = GA (wk) ±2, for pregnancy 20–36 weeks. There was a strong statistical evidence that our data fitted more appropriately with a quadratic pattern (*P* < 0.001 from likelihood-ratio test).

However, one should be aware that fundal height measurement is more or less subjective to error, either from intra- or interobservers. To minimize such limitation, standardization and regular calibration should be emphasized.

Being a retrospective data collection, some of the data were inevitably incomplete. Our study, however, tried to collect a large sample sized data to allow for missing values.

Like other clinical prediction rules, the derived fundal height growth curve should be validated before putting into routine clinical practice. 

## 5. Conclusions

A demographically specific fundal height growth curve is a simple tool for monitoring intrauterine growth and screening for abnormal uterine growth. Applying fundal height growth curve into routine antenatal care practice may reduce unnecessary ultrasound in fully equipped settings and reduce unnecessary referring for further investigations in resource-deprived settings.

## Figures and Tables

**Table 1:** Characteristics of study subjects (*n* = 1,038).

Characteristics	*N*	%
Settings		
Secondary care hospitals	696	67.0
Tertiary care hospitals	342	33.0
Maternal age (year) (mean, ±SD)	25.6	±6.2
Maternal height (cm) (mean, ±SD)	156.1	±5.6
Before pregnancy weight (kg) (mean, ±SD)	52.6	±9.8
Before pregnancy BMI (kg/m^2^) (mean, ±SD)	21.6	±3.8
Total weight gain (kg) (mean, ±SD)	13.5	±4.7
Parity		
Nulliparous	523	50.4
Multiparous	515	49.6
GA at first antenatal visit (wk) (mean, ±SD)	13.0	±5.0
GA at delivery (wk) (mean, ±SD)	39.2	±1.1
Type of delivery		
Normal	766	73.8
Cesarean	236	22.7
Vacuum	32	3.1
Forceps	4	0.4
Infant's sex		
Female	479	46.2
Male	559	53.8
Birth weight (gm) (mean, ±SD)	3,120.3	±325.0

GA: gestational age; SD: standard deviation.

**Table 2:** Mean and standard deviation of fundal height in centimeters for each gestational age based on 1,038 normal singleton pregnancies (7,523 visits).

GA (wk)	Number of measurement	Fundal height (cm)	
		Mean	SD
20	166	19.1	1.9
21	144	20.3	1.8
22	119	21.5	2.1
23	123	22.4	1.8
24	266	23.7	1.7
25	235	24.4	1.8
26	126	25.3	1.7
27	142	26.6	1.7
28	349	27.7	1.8
29	268	28.6	1.8
30	352	29.7	1.7
31	336	30.4	1.7
32	438	31.5	1.6
33	386	32.2	1.7
34	409	33.1	1.6
35	391	33.8	1.7
36	473	34.4	1.7
37	739	34.9	1.9
38	840	35.0	2.0
39	762	35.2	2.3
40	459	35.4	2.4

GA: gestational age; SD: standard deviation.

**Table 3:** Fundal height at the 10th, 50th, and 90th percentiles in centimeters between 20 and 40 weeks of gestation, derived from a quadratic regression model.

GA (wk)	Number of measurements	Fundal height (cm)		
		Percentiles		
		10th	50th	90th
20	166	16.2	18.7	21.1
21	144	17.6	19.9	22.3
22	119	19.1	21.3	23.6
23	123	20.4	22.5	24.7
24	266	21.7	23.7	25.9
25	235	22.8	24.7	26.8
26	126	24.0	25.9	27.9
27	142	25.1	26.9	28.9
28	349	26.1	28.0	30.0
29	268	26.9	28.8	30.7
30	352	28.0	29.8	31.8
31	336	28.6	30.5	32.5
32	438	29.5	31.4	33.5
33	386	30.1	32.0	34.2
34	409	30.8	32.8	35.0
35	391	31.2	33.4	35.6
36	473	31.8	34.0	36.4
37	739	32.2	34.5	37.0
38	840	32.5	35.0	37.6
39	762	32.8	35.4	38.1
40	459	33.0	35.8	38.6

GA: gestational age.

**Table 4:** Fundal height growth curves from existing studies.

Source	Country	Number of women	Number of visits	LMP/US	Subjects	GA (wk) and FH (cm)					
						20	24	28	32	36	40
Calvert et al., 1982 [18]	UK	313	1,775	LMP	Normal	18.8	22.9	26.8	30.2	33.7	36.2
Linasmita and Sugkraroek, 1984 [16]	Thailand	415	1,295	LMP	Normal	17.7	23.1	26.4	30.4	32.9	34.6
Ngan et al., 1988 [19]	Hong Kong	—	1,051	LMP	—	17.9	22.0	25.9	29.5	32.8	36.1
Rai et al., 1995 [20]	India	100	523	LMP	Normal	18.9	22.8	26.9	31.0	34.4	37.3
Hakansson et al., 1995 [12]	Sweden	403	4,189	LMP and US	Normal	19.0	23.0	27.0	30.5	33.5	35.5
Walraven et al., 1995 [13]	Tanzania	83	403	LMP	Normal	16.2	20.3	23.6	27.8	31.2	33.6
Limpanyalert and Manotaya, 2001 [17]	Thailand	199	879	US	Normal	20.1	24.2	28.7	32.0	35.8	36.9
Challis et al., 2002 [14]	Mozambique	817	6,544	US	All	19.0	23.0	26.8	30.0	33.0	35.0
Mador et al., 2010 [15]	Nigeria	405	405	US	—	19.1	24.4	28.3	32.0	35.8	39.3
This study, 2012	Thailand	1,038	7,523	LMP and US	Normal	18.6	23.7	27.9	31.4	34.0	35.8

GA: Gestational age; FH: fundal height; LMP: GA by last menstrual period; US: GA by ultrasound.
